# Ion beam nanopatterning of III-V semiconductors: consistency of experimental and simulation trends within a chemistry-driven theory

**DOI:** 10.1038/srep18207

**Published:** 2015-12-16

**Authors:** O. El-Atwani, S. A. Norris, K. Ludwig, S. Gonderman, J. P. Allain

**Affiliations:** 1 School of Nuclear Engineering, Purdue University, West Lafayette, IN 47907; 2 School of Materials Engineering, Purdue University, West Lafayette, IN 47907; 3 Birck Nanotechnology Center, West Lafayette, IN 47907; 4 Department of Mathematics, Southern Methodist University, Dallas, TX75275; 5 Physics Department and Division of Materials Science and Engineering, Boston University, Boston, Massachusetts, 02215, USA; 6 Department of Nuclear, Plasma and Radiological Engineering, University of Illinois at Urbana-Champaign, Urbana, IL, 61801

## Abstract

Several proposed mechanisms and theoretical models exist concerning nanostructure evolution on III-V semiconductors (particularly GaSb) via ion beam irradiation. However, making quantitative contact between experiment on the one hand and model-parameter dependent predictions from different theories on the other is usually difficult. In this study, we take a different approach and provide an experimental investigation with a range of targets (GaSb, GaAs, GaP) and ion species (Ne, Ar, Kr, Xe) to determine new parametric trends regarding nanostructure evolution. Concurrently, atomistic simulations using binary collision approximation over the same ion/target combinations were performed to determine parametric trends on several quantities related to existing model. A comparison of experimental and numerical trends reveals that the two are broadly consistent under the assumption that instabilities are driven by chemical instability based on phase separation. Furthermore, the atomistic simulations and a survey of material thermodynamic properties suggest that a plausible microscopic mechanism for this process is an ion-enhanced mobility associated with energy deposition by collision cascades.

Over the last two decades, the formation of ordered surface nanostructures via low-energy broad ion-beam irradiation has been studied as a bottom-up, fully parallel nanofabrication technique^1^. Ripple-like structures have long been observed under oblique incidence irradiation of glasses and silicon^2^,^3^, and more recently ordered nanodots have been observed under normal incidence irradiation of III-V compound semiconductors^4^ or group IV semiconductors laced with silicide-forming impurities^5^,^6^,^7^,^8^,^9^,^10^. These latter structures are of great technological interest, and it seems clear that they are correlated to the presence of distinct and compound-forming atoms in the target^7^,^8^,^11^. It remains crucial, however, to understand the physical mechanism responsible for the formation process to correlate nanostructure characteristic parameters (characteristic length, aspect ratio, size, order) with irradiation conditions before qualifying the method as a scalable and reproducible nanofabrication process.

Several groups^12^,^13^,^14^,^15^ have performed experimental studies on how ion-beam irradiation parameters such as ion energy, flux, fluence, temperature and incident ion angle affect the nanostructure shape and size (e.g. shallow nanodots^4^ versus steep pillars^12^) on multi-component systems (e.g. in particular with III-V irradiation). Surface compositional effects were also experimentally studied^16^,^17^,^18^,^19^. El-Atwani *et al.*^18^ studied how the surface composition changes with the irradiation fluence (before and after the formation of the nanostructures) using *in-situ* x-ray photoelectron spectroscopy (XPS) and low energy ion scattering spectroscopy (LEISS) during irradiation of GaSb surfaces (with and without native oxides) at different ion energies including the sputter threshold regime^17^.

On the other hand, several attempts have emerged in the literature to theoretically model the initial instability that ultimately leads to III-V compound semiconductor nanostructure formation using linear theory. These linear theories would apply only at the very beginning of the nanostructure formation process; they do not attempt to describe the complex structures ultimately formed. Building on the early model of two-component surfaces by Shenoy, Chan, and Chason^20^, Bradley and Shipman showed how preferential mass redistribution could mediate the effect of curvature-dependent erosion to produce ordered structures^21^,^22^. In contrast to this morphological instability, an alternate driving mechanism based on a chemical instability was proposed by Le Roy *et al.*^12^, who proposed Ga segregation and Ga mask formation during early irradiation stages. Norris^23^ demonstrated that this mechanism also contained the ingredients needed to produce ordered structures.

However, although there has been considerable effort to experimentally identify the dependence of pattern formation on the irradiation environment, and to theoretically model the potentially relevant mechanisms, there has been only modest success in connecting experiment to theory for the purposes of testing and validation. This is primarily due to the difficulty of directly connecting current experimental results on nanopatterning and compositional variation to existing computational and theoretical model parameter space. Furthermore, the disparate time scales between experiments (e.g. time scale of probes ranging from 0.1 sec to 1000’s sec) and prompt atomistic computational models (e.g. picoseconds up to nanoseconds) also makes it challenging to make these direct links. On the other hand, phenomenological models that can successfully model pattern formation are hindered by the use of unknown experimental terms and coefficients that are simply varied to achieve a desired pattern.

In this study, we take a different approach. The experimental study of a range of targets (GaP, GaAs, GaSb) and ion species (Ne, Ar, Kr, Xe) allows the experimental determination of parametric trends in the presence or absence of patterns with varying ion and target mass. In tandem, we have performed atomistic simulations using the Binary Collision Approximation over the same range of ion/target combinations, observing trends in statistical quantities relevant to each model. By comparing the trends in the experiments to the trends in the simulations through the lens of each model in turn, we can identify correlations that point to fundamental physics. Ultimately, our findings suggest that the experimental and simulation trends are broadly consistent within the model focusing on phase-separation, if the latter is assumed to be enabled by an ion-enhanced mobility induced by energy deposition within the collision cascade.

## Methods

### Experimental Methods

Irradiation of III-V samples was performed in the Particle and Radiation Interaction of Hard and Soft Matter (PRIHSM) facility at Purdue University. A gridded, non-reactive ion source (Tectra Gen II)was used to irradiate the samples. Irradiation was performed at normal incidence using Ne, Ar, Kr and Xe ions with 500 eV ion energy at a flux of 2 × 10^14^.cm^−2^.s^−1^. Temperature was kept near room temperature via the combination of a resistive heater and liquid nitrogen cooling.

Morphology investigation was performed in the Birck Nanotechnology Center (BNC) at Purdue University. A high-resolution scanning electron microscope (HRSEM) was used. In addition, real time grazing incidence small angle x-ray scattering (GISAXS)/irradiation studies were performed on beam line X21 of the National Synchrotron Light Source (NSLS) at Brookhaven National Laboratory (BNL). The GISAXS incident x-ray angle was 0.8° and the exit angle was 0.2°, while the x-ray energy was 12 keV. The operating pressure was around 5 × 10^−7^ torr. Irradiation was performed using the same source described above with similar energy and ion flux.

Finally, XPS and LEISS characterization were performed. For XPS characterization, a Mg Kα (1253.4 eV) excitation source was used. For LEISS characterization, the sample was irradiated at an angle of 55° relative to the normal, with a backscattering angle of 145°. Helium ions were used for LEISS characterization of GaSb and GaP, while for GaAs, Neon ions were used. The energy and the flux of the ions were 1500 eV and 1–3 × 10^13^.cm^−2^.s^−1^ respectively. Operating pressure was 2 × 10^−8^ torr and the partial pressure of oxygen was under 9 × 10^−11^ torr as read from the residual gas analyzer (RGA). Both LEISS and XPS were performed *in situ* with no atmospheric exposure after irradiation to inhibit any segregation effect of one component to the surface due to oxide formation^15^ and to ensure no contamination occur from the ion source or the sample holder.

In this work we operate the Tectra Gen II broad-beam ion source with a molybdenum 3-grid configuration. Ludwig and co-workers demonstrated the importance of Mo impurities during nanodot formation on silicon substrates via ion beam irradiation^5^,^6^,^24^. Subsequently, El-Atwani *et al.*^15^,^17^,^18^ established with *in-situ* surface chemistry studies during nanopatterning of silicon via ion beam irradiation that the concentration of different impurities needed to induce nanostructure formation by this mechanism is between 2–5 atomic %. In this study, the current in the extractor is minimized to minimize sputtering from the grid, and we found that irradiation at energies over 200 eV induces no detectable Mo impurities, as measured *in-situ* by LEISS and XPS. Typical sensitivies of these techniques are between 0.1–0.5 atomic %, well below the threshold needed to induce impurity-driven nanopattern formation in previous studies.

### Simulation Methods

Atomistic Simulations were performed using the Binary Collision Approximation (BCA) code TRI3DST^25^. Because BCA codes treat the collision cascade as a branching series of purely repulsive binary atomic interactions, additional energetic inputs must be supplied, which have important effects on the behavior of the simulations. For all atomic species, we have followed recommended conventions in the TRI3DST documentation (which accompanies the software) by choosing a bulk binding energy of 0 eV, a displacement threshold energy of 5 eV, and a cutoff energy of 1 eV. The last energy to be specified is the surface binding energy. Here we use a standard linear concentration-dependence of the form


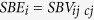


where 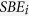 is the surface binding energy of atoms of species 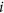, and 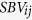 is a matrix relating the values of these surface energies to the concentrations 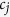 of each species. As described in the documentation, the diagonal elements of this matrix can be chosen using the enthalpy of sublimation


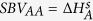



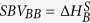


while conservation of energy considerations lead to a choice for the off-diagonal elements of


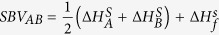


where 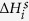 is the enthalpy of sublimation of species *i*, and 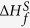 is the enthalpy of formation of the compound. In principle this approximation is only valid near the 50/50 concentration of the virgin compound, and may break down as the concentration changes under ion irradiation; however, more rigorous nonlinear models are not readily available.

In addition to interaction energies, the output of simulations depends of course upon the properties of the irradiated target. In principle, the target should have a concentration profile to match the steady-state profile of the irradiated material^23^, which can deviate from a homogeneous 50/50 composition due to effects such as preferential sputtering and redistribution, ion-induced mixing, and Gibbsian segregation^26^. However, the steady profiles are not known experimentally, and not all of these effects can be captured within the timescale associated with the simulations, rendering atomistic estimation unavailable. Because these data are not presently known experimentally, we perform simulations over a range of homogeneous target compositions ranging from 20/80 to 80/20.

The conversion of simulation statistics to the coefficients of partial differential equations relies upon the crater functions framework, introduced in generic form in refs 27,28 and particularized to various sets of physical assumptions in refs 29,30. The methods used within this paper are based upon methods for binary materials described in ref. 31, but equations (16) in that paper are modified by the addition of terms identified in ref. 30 to describe explicit curvature-dependence of the impact statistics. In addition, whereas derivatives with respect to concentration were estimated via interpolation in ref. 31, here they are calculated via finite differences applied to simulations at different target concentrations. All results are obtained using the recently-introduced PyCraters library^23^, which provides a simple user interface for the performance of calculations of this type.

## Experimental Results

**Figure 1:**
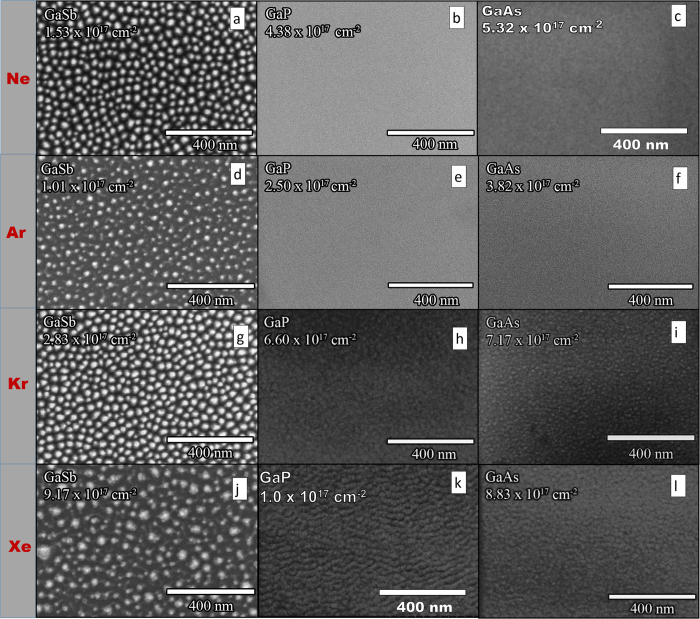
SEM micrographs of irradiated III-V semiconductor targets (GaSb, GaP, GaAs) with 500 eV energetic ions (Ne, Ar, Kr and Xe).

For morphology investigation, GaSb, GaAs, and GaP surfaces were irradiated with 500 eV Ne, Kr, Ar and Xe. For possible nanopattern formation on the semiconductor surfaces, the irradiation fluence (ion dose) was changed. It should be noted that irradiation parameters were similar to those used in previous studies^12^,^15^,^17^,^32^,^33^. It was found that for GaSb samples, irradiation with every ion type induced nanostructure formation. For GaAs and GaP, however, only heavy ions (Kr and Xe) led to nanopattern formation on the irradiated surfaces. These results are summarized in Fig. 1, which contains SEM images of samples at the end of the irradiation process.

Since different semiconductors can have different fluence threshold for nanostructure formation, real time GISAXS/irradiation was performed to rule out possible fluence effects on the irradiated surface by observing the sample irradiation response in real time. Within a kinematical scattering approximation, in a GISAXS scan, the relation between the height of the surface/nanostructures, h(x, y) and the intensity is described in the following equation^34^:


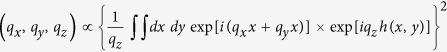


If 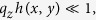 then the intensity of the GISAXS scan is proportional to the magnitude of the Fourier transform of 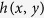 squared. The wavenumbers, 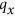 and 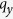 are equivalent and therefore, they are described by 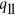. The *q*_ll_ = 0 peak is the near-specular peak and satellite peaks on each side of the specular peak indicate nanostructure formation with a real space distance of 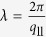
35. While GISAXS is a very sensitive technique to nanostructure height, when structures grow sufficiently high that the small-height restriction above is violated, interference between the top and bottom of structures leads to a smearing of the intensity so that GISAXS intensity peaks are washed out even if well-defined correlated nanostructures persist^36^.

**Figure 2:**
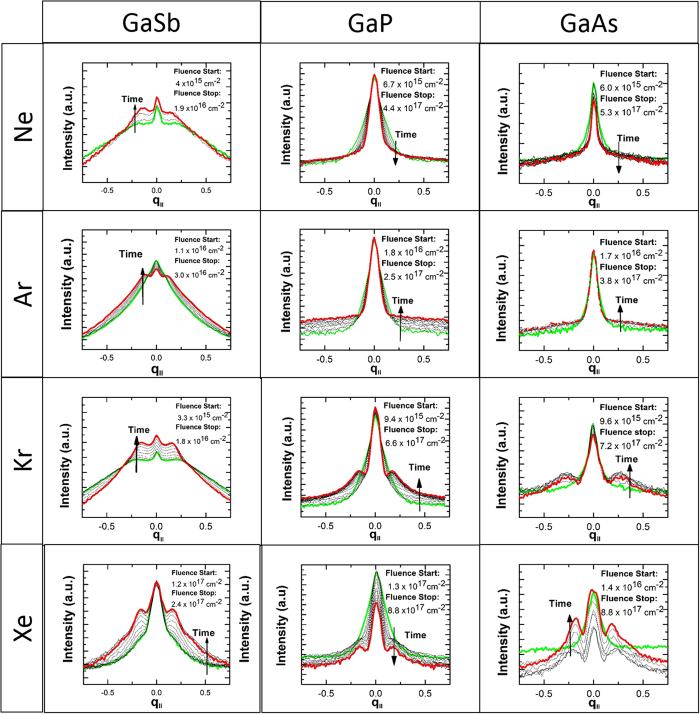
Real time GISAXS spectra obtained during irradiation of III-V semiconductor targets (GaSb, GaP, GaAs) with 500 eV energetic ions (Ne, Ar, Kr, Xe). For every target, the GISAXS spectra was plotted at the same fluences for better comparison. Initial (green) and final (red) fluences are chosen to best highlight satellite peak formation for GaP and GaAs under Kr and Xe irradiation. Under Ne and Ar irradiation, the spectra for these materials show smoothing instead. Finally, for GaSb, the pattern had emerged already by the time the GISAXS measurements began, so only the initial spectra is presented. At later times, the GaSb peaks become washed out due to large amplitudes of the patterns, and the consequential destructive interferences in the scattered x-rays, which can remove well-defined peaks^36^.

The GISAXS experiments were performed up to a fluence of 5–8 × 10^17^ cm^−2^, and Fig. 2 shows the GISAXS spectra at various times throughout the irradiation process for every ion-semiconductor combination. The results are entirely consistent with the SEM images obtained after irradiation in PRIHSM when it is kept in mind that correlation peaks are smeared out when structures become tall (i.e. *q*_*z *_*h* ≥ 1). Correlated nanostructure formation is indicated by the satellite peaks in the GISAXS spectra and confirmed by SEM images of GaSb regardless of the ion type. On GaAs and GaP, however, the GISAXS/irradiation and the irradiation/SEM characterization experiments showed only nanopattern formation using Kr and Xe. Furthermore, the satellite peaks had 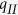
values of 0.18–0.25 nm^−1^ which correspond to ~25–35 nm distance between the nanostructures, in agreement with the SEM images.

**Figure 3:**
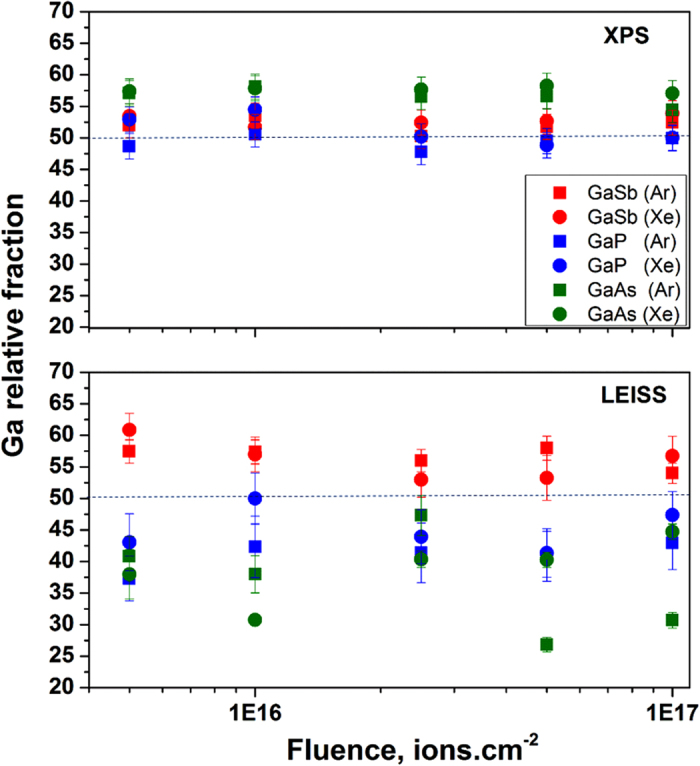
Gallium relative fraction as a function of fluence determined from *in-situ* XPS and LEISS measurements during 500 eV ion (Ar, Xe) irradiation on III-V semiconductor targets (GaSb, GaP).

To investigate surface compositional effects on the nanopatterning process and the above results, XPS and LEISS were performed on the semiconductor surfaces using two ion types: Ar and Xe. Since Ar irradiation on GaAs and GaP surfaces does not form nanostructures while Xe does, XPS and LEISS studies using these two ion species are thought to demonstrate any possible surface composition/sputtering dependence. Every *in-situ* irradiation/(LEISS + XPS) experiment was performed several times. Results of these measurements are shown in Fig. 3. It should be noted that in the case of Ar and Xe irradiation on GaAs, Ga and As peaks overlap and peak fitting was necessary to resolve them. The fitting was done using Igor software and the corresponding error (error bar) is given. Therefore, some fluctuations may arise in the LEISS data of GaAs.

The difference in the results between the XPS and the LEISS is consistent with the different depths probed by the two techniques. Because low binding energy peaks were quantitatively measured, XPS can probe ~8 nm from the surface – i.e., it observes an average over the entire amorphous film – while LEISS probes the first monolayer only^37^. Therefore, different results from the two techniques indicates non-constant vertical composition profile. Enrichment of one component (more obvious in the LEISS results) is a sign of surface composition changes during the course of irradiation, which would happen due to preferential sputtering or segregation effects. The results are independent of the ion type, and both Ar and Xe irradiation cases show similar component enrichment (Fig. 3). More discussion on these topics appears elsewhere^17^.

### Model Comparison and Discussion

#### Background: Single-component models

The first theory on pattern formation during ion sputtering is due to Sigmund^38^, who noted that under an idealized model of the collision cascade due to ion impact, a higher relative sputter rate was expected in concave regions compared to convex regions, which produces a morphological instability. This observation was quantified by Bradley and Harper^39^, whose stability analysis enabled the first predictions on pattern selection and wavelength. Later, it was noted that a weakly-nonlinear version of the model yielded, to leading order, the Kuramoto-Sivashinsky equation^40^, and many subsequent works proposed modified versions of this equation^41^. These have included a linear “damped” version^42^ an “extended” version with an extra nonlinear term^43^. Even more recently, the effect of long-range redeposition of sputtered atoms has been considered, which produces a nonlocal effect^44^,^45^,^46^ that is also nonlinear^47^. For normal incidence irradiation, these results may be summarized in the single equation


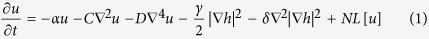


Here the terms 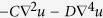 appeared in the original Bradely-Harper model, the lowest-order nonlinearity 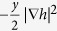 appeared in ref. 40the linear damping term 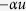 appeared in ref. 42and the additional nonlinear term 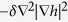 appared in ref. 43 to describe arrested coarsening. Finally, the term 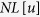 describes the (nonlocal) effect of long-range atomic redeposition considered in refs 44, 45, 46.

Equation (1) shows that all these models share two important features: (1) they describe only pure materials, and (2) they assume a basic instability driven by erosion, with a negative value of the coefficient 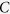 in front of the 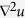 term. More recent results in the field, however, have indicated the need for a more flexible approach. Recent experimental^48^,^49^ and atomistic^29^ studies have suggested that in this energy regime, the erosive instability is typically suppressed at normal incidence by the effect of atoms merely redistributed by the impact, which leads to a positive value of 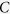, especially near normal incidence. Concurrently, experimental results have pointed to the importance of a second component in the irradiated target, showing that patterns on pure surfaces can be eliminated^5^ or induced^6^ by the careful removal or addition of impurities. This strongly suggests that a more general approach incorporating concentration effects is needed.

### Two-Component Models

In light of the historical context just given, we limit our attention for the remainder of the paper to two recent descriptions of surface nanopattern formation in two-component systems. Conveniently, both mechanisms can be incorporated into a common model with the linearized form


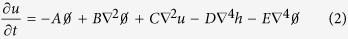



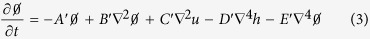


These equations – presumably valid at the very early stages of pattern formation – describe the time evolution of fluctuations 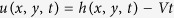 of a height field away from a flat interface eroding with speed V, and 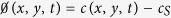 of a concentration field away from its steady-state value. The precise meaning of all parameters is documented extensively elsewhere, but for narrative purposes we here omit a lengthy set of definitions, and instead give brief, qualitative descriptions of each term. We refer the reader to refs 20, 21, 22, 23,50 for more details.

The coefficients 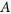 and 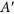 are both associated with the concentration dependence of the sputter yield. The term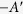 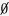 in Eq. (3) is perhaps most easily understood–for any stable steady concentration 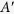 is positive and this term therefore serves as a restoring mechanism. If, say, the fraction of A atoms increases from the steady value, then the sputter rate of A atoms will increase and that of B atoms will decrease, pushing the system back toward steady state in the absense of other effects. The term −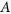 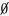 in Eq. (2) is somewhat more subtle–it describes a change in the total sputtering rate as the concentration strays from the steady value. This depends on a difference of the slopes of the sputter yields with respect to concentration, and can be made positive by choice of the atomic species represented by the dimensionless concentration fraction 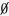 (see ref. 23).

The coefficients 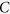 and 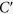 are associated with curvature-dependence of the collision cascade, through the competing mechanisms of curvature-dependent erosion and lateral mass redistribution. In Eq. (2), the term 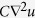 represents the sum of these effects across both species–if 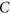 is positive, this is a stabilizing effect, while if 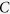 is negative, then it represents a destabilizing morphological instability in the height field. The counterpart of this term, 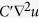 in Eq. (3) describes how differences between species in these effects can change the concentration field. For instance, if the ion-driven downhill redistributive fluxes are different between species, then gradients in the height field can lead to changes in the concentration.

The coefficients 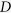 and D′ are primarily associated with the dissipation of surface energy through fluxes proportional to gradients of curvature. These can occur, for example, via surface self-diffusion, or via surface-confined viscous flow, under which mechanisms the coefficient 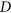 is always positive, and the term −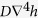 in Eq. (2) therefore serves to regularize the equations in the case that 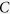 is negative. If the mobilities of the two species are different, then gradients in the curvature can produce different fluxes of the two species, leading to changes in the concentration via the term 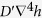 in Eq. (3). Additional contributions to these terms could come from high-order expansions of the statistics of the collision cascade (see for example refs 21,41).

The coefficients B, B′, E, E′are primarily associated with the dissipation of chemical energy through fluxes proportional to gradients of chemical potential. The terms 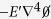 and 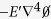 in Eq. (3) appear following a standard variational analysis of a potential energy depending both on concentration and the square of its gradient. If the potential is non-convex in the concentration, then B′ < 0, and the term 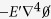 is needed to keep the equation for the concentration field well-posed. Finally, if the mobilities of the two species are different, then diffusion down chemical potential gradients can cause changes in the height field, producing the corresponding terms 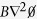 and 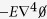 in Eq. (2). Again, additional contributions to all of these terms can in principle come from expansions of the statistics of the collision cascade (see for example ref. 23).

### Two Routes to Instability

Mathematically, the stability of field equations is expressed in the *dispersion relation* 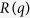, specifying the growth rate of sinusoidal perturbations with wavenumber 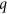. For Eqs (2,3), the dispersion relation is obtained by means of a matrix containing 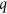 and the coefficients therein. Given the apparently stationary nature of the structures, it can be reasonably assumed that stability is governed by the sign of the determinant 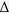 of this matrix. Following historical precedent by neglecting the coefficients 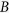 and 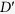, one obtains


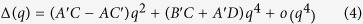


with negative sign leading to an instability and positive sign leading to stability (the higher-order terms are assumed positive so that very short wavelengths are always stable). Moreover, it is often found that patterns on III-V semiconductors are fairly monodisperse, with a narrow range of wavenumbers present in the surface structure. Such an observation suggests that 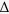 is only negative over a finite range of 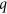, that in particular excludes 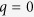. (In the language of ref. 51, this is a “Type I” instability). This behavior of 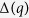 can only occur if both

1. *A*′*C* − *AC*′ > 0 (i.e., the long wavelengths are stable)
2. *B*′*C* < −*A*′*D* (either *C* or *B*′ are sufficiently negative)

We note that it is not sufficient for 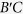 simply to be negative in Eq. (4) – rather, it must be *sufficiently* negative to overcome the stabilizing tendencies of the restoration coefficient 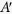 and the surface-energy reduction coefficient 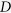, both of which are positive.

As just observed and also discussed elsewhere^21^,^23^, there are at least two very distinct mechanisms that could produce such a functional structure – a morphological instability caused by a negative value of 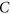, and a chemical instability caused by a negative value of 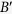. Unfortunately, although methods have been developed to estimate some of the parameters appearing above via atomistic simulation^31^, others of the parameters represent diffusive mechanisms that operate on timescales too long to simulate. In addition, direct experimental observation of potential signatures distinguishing the regimes^23^ is hampered by the need of *in-situ* observations^15^,^18^.

In light of these limitations, we here take a different approach. The existence of experimental observations of a *range* of both ion species and target compounds provides a unique opportunity to compare models not just to a single experiment, but to experimental *trends*. It is often the case that a proffered model can reproduce a particular experimental result for an appropriate choice of the model’s parameter values; however, if the model is consistent with a range of such results, confidence in its faithfulness to the underlying physical system is increased. Therefore, we here compare the predictions of both models with simulation data on parameters themselves, or related proxies, to two trends in the experimental observations

1. An apparent increase in surface instability for higher ion masses.
2. An apparent increase in instability for GaSb relative to GaAs and GaP.

### Morphological Instability: Trends in C

The first instability mechanism is a variant of the Bradley-Harper instability for pure materials^39^, where the curvature-dependent sputtering mechanism identified by Sigmund^38^ was shown to produce, to leading order, the term 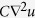 in Eq. (2), with an unconditionally negative value of 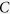. For a pure target, this term leads to a morphological instability at all wavenumbers 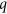, which is then regularized at high wavenumbers by the inclusion of a higher-order stabilizing mechanism such as surface diffusion^52^ or surface-confined viscous flow^53^ both of which, when linearized, yield terms of the form 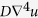 in Eq. (2) (see ref. 39). Finally, if a second target component is added, it is possible for a positive value of the parameter group *A*′*C−AC*′ to stabilize the small wavenumbers (large wavelengths), leaving a narrow band of unstable wavenumbers 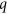
21,22.

**Figure 4:**
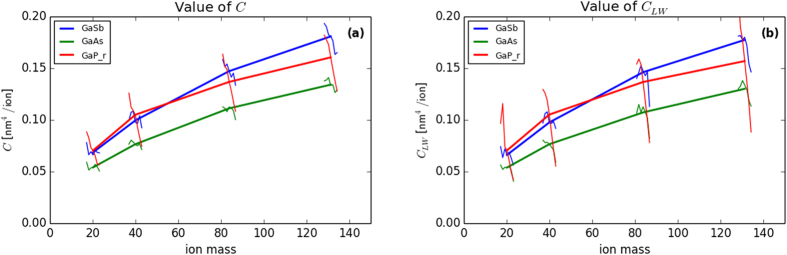
Shown are estimates of (**a**) the coefficient 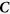 describing the competition between sputter erosion and mass redistribution, (**b**) the coefficient group 
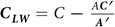
, in units appropriate to single ion impacts (i.e., the values must be multiplied by the ion flux to convert to the usual units of nm^2^/sec.). Each quantity is presented as a function of ion mass (ion type) for each of the three targets used in this study, for compositions ranging from 80/20 to 20/80. The circles connected by thick lines indicate the median of these estimates, while the thin lines at each data point represent the ranges over composition.

Because it is built upon the classical Bradley-Harper instability for pure materials, this mechanism requires a sufficiently negative value of 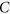, which is clearly destabilizing to the height field if we were to set 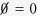. In ref. 21, the coefficient 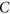 is defined via


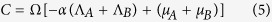


where 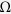 is the average atomic volume, 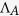 and 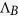 describe the sputter yields of A and B species, and 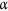 is a proportionality constant. In addition, 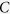 contains contributions 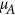 and 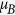 from the competing effects of collision cascade-induced ballistic displacements identified by Carter and Vishnyakov^54^. These contributions can in principle change this constant to a positive value^29^,^55^, highlighting a competition within the coefficient 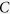 between the destabilizing (at normal incidence) curvature-dependent sputtering mechanism and the stabilizing (at normal incidence) ballistic displacement mechanism. Together, these mechanisms capture most effects of the collision cascade on the height evolution. Conveniently, the individual parameters in Eq. (5) can be estimated from atomistic simulations using methods described above; results are reported in Fig. 4a,b.

The most immediate result is that *C* is observed to be positive for all ion/target combinations over a broad range of potential steady surface concentrations 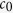, and becomes increasingly positive as the ion mass increases. This is consistent with observations on irradiated Si at similar energies using molecular dynamics^29^,^31^. If the morphological instability due to curvature-dependent sputtering were the cause of the nanostructures in Fig. 1, we would expect 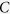 to be *negative* for most ion/target combinations, and to *decrease* with the ion mass, to explain the emergence of patterns at high ion masses for GaAs and GaP.

### Long wavelengths: trends in *A*′*C* − *AC* 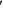


For completeness, we also estimate the sign of the parameter group *A*′*C−AC*′ which, if negative, can also produce an instability (though not one leading to monodisperse structures)^23^,^56^,^57^. More specifically, we estimate the value of


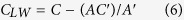


which appears as the first term in the Maclaurin series of 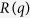. The results are broadly the same as preliminary estimates for Ar → GaSb in ref. 31, with the first term in this group having a far larger value than the second, leading to essentially the same values for 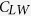 as for 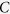 itself. Hence, the behavior of long wavelengths seems dominated by stabilizing redistributive effects, and increasing ion mass increases the stabilization. We conclude that if only ballistic effects due to collision cascades are considered, then under normal-incidence ion irradiation, all of the ion-target combinations we have considered would be expected to exhibit stable, featureless surfaces. It is instructive to note that this is exactly the behavior seen on pure group IV semiconductors such as Si^48^ and Ge^49^, in which the ballistic mechanism is the only one available.

### Chemical Instability: Trends in 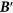


An alternate instability mechanism has been proposed known as self-sustained etch masking^12^,^58^ in which structures appear not from a morphological instability in the height field caused by the cumulative effect of collision cascades, but from a chemical instability in the concentration field over much longer timescales, as described by the Cahn-Hilliard equation^59^. The proposed sequence of events is that first, preferential sputtering of one of the two elements leads to a thin film at the surface of the target with a steady concentration 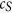 that is different than 50%, as we observed for each target material in Fig. 3 (note that the preferential sputtering, itself, can be driven by ion-induced modification of the near-surface concentration profile^26^,^60^). Given the line compound in the III-V phase diagram, and the fact that experiments are conducted at room temperature, this is likely a concentration at which the energetics of the problem favor spinodal decomposition, into regions of the 50/50 line compound, and regions nearly pure in the less-sputtered element. Finally, a difference in the sputter yield of these two *phases* (captured in the term −*A*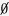 of Eq. (2), and distinct though related to the preferential sputtering between *species* invoked above) induces an accompanying modification of the height field.

Because it is built on the classical Cahn-Hilliard instability, this mechanism requires a sufficiently negative value of 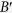, which is clearly destabilizing to the chemical concentration field if we were to set 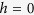. In ref. 23the coefficient 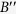 is defined via


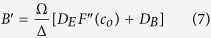


where 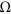 is again the average target atomic volume, 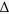 is the amorphous film thickness, 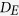 is an energetic mobility, 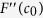 describes the (assumed negative) second derivative of the free energy curve at the steady-state concentration, and 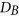 is an unconditionally-positive contribution describing ballistic diffusion. Now, the quantity 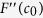 is predominantly a property of the target rather than the ion mass, so that the ion-dependence of of 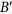 depends mostly on the relative sizes of the energetic and ballistic diffusivities 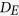 and 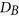. We now consider each component in turn, to the extent admitted by an atomistic study, and in the process we shall make two important assumptions.

### Ballistic Diffusivity

The ballistic diffusivity can be estimated directly from collision cascades – it is defined to be^23^ :


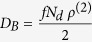


**Figure 5:**
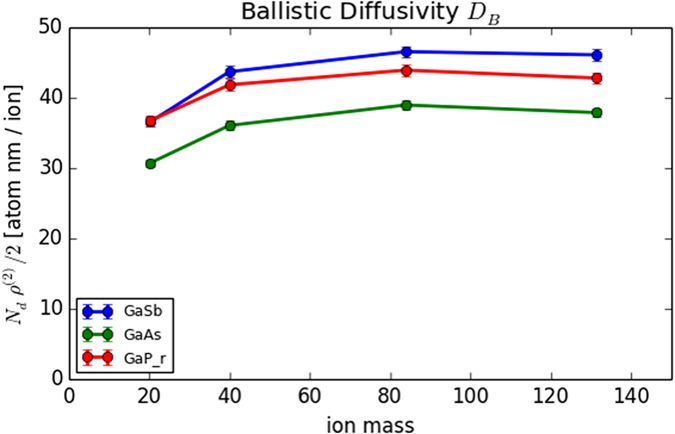
Shown are estimates of the ballistic diffusional mobility 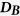, presented as a function of ion mass (ion type) for each of the three targets used in this study. The dependence is weak – after an initial increase from Neon to Argon irradiation, further mass dependence in not observed.

Where 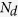 is the number of displacements per ion impact, and 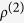 is the second moment of the displacement length distribution. These quantities are recorded by default in the PyCraters library, and allows us to obtain the estimates of 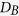 shown in Fig. 5. There we see only a weak trend of slightly increasing 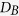 as a function of ion mass. If ballistic diffusion were a dominant effect, we would expect slightly increased stabilization of surfaces as the ion mass increases, which is not the observed behavior. Therefore, our first assumption is that the (stabilizing) effect of ballistic mixing is small compared to the (destabilizing) effect of phase separation, so that


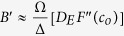


This assumption seems reasonable in light of studies of film *growth*, where surface-confined phase separation of immiscible materials is readily observed during both thermal^61^,^62^ and hyperthermal^63^ deposition conditions. Because hyperthermal deposition produces collision cascades similar to those occurring during sputter erosion, and the ballistic mixing induced by those cascades does not appear to suppress phase separation, we will therefore assume that 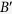 is dominated by energetic effects, and confine inquiry to the first term in Eq. (7), describing diffusion along gradients of the chemical potential.

### Diffusional Mobility

Our second assumption, introduced previously^23^, is that *the diffusional mobility is enhanced by the ion beam*, so as to correlate with a proxy statistic associated with the collision cascade. This assumption is similar in spirit to models based on ion-enhanced fluidity^53^,^64^, and has long been applied to crystalline materials under ion irradiation^65^,^66^. Mobility enhancement has been observed directly via molecular dynamics simulation^67^ and has also been invoked to explain banding observed during film growth^68^.

**Figure 6:**
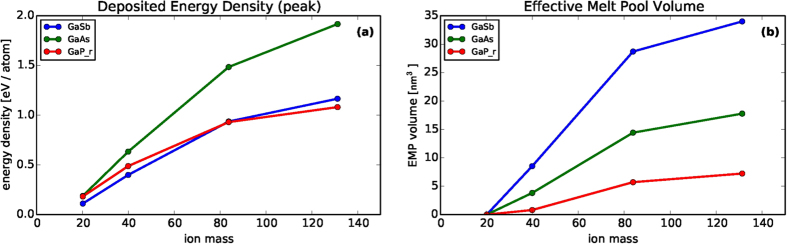
Shown are estimates of (**a**) the peak value of the deposited energy density (at the center of the collision cascade), and (**b**) the volume of “effective melt pools” due to energy deposition, as given by Eq. (8). Each quantity is presented as a function of ion mass (ion type) for each of the three targets used in this study.

As an observable proxy for the enhancement to mobility, we will here consider the deposited energy density, under the reasoning that energy supplied by the ion beam during the collision cascade may supply target atoms with the energy needed to cross saddle points in the local energy landscape, enabling them to more readily reduce their free energy by moving to more energetically-favorable locations. The energy density has been estimated for each ion/target combination by dividing 500 eV by the volume of an ellipsoid with major and minor radii equal to twice the standard deviation of lateral and longitudinal straggle in the distribution of ion implantation locations. These values are reported in Fig. 6a. A clear trend of increasing energy density with ion mass is observed. Under the above assumptions this would be increasingly destabilizing as the ion mass is increased, which is in fact the observed behavior.

### Connection with material thermal properties: Effective Melt Pools

We are given pause by the observation that its large collision volumes give GaSb the lowest deposited energy density, which at first seems inconsistent with the fact that structures develop fastest and most strongly for this material. However, GaSb has several mitigating properties that offset its large collision size. Specifically, compared to the other two targets, it has the lowest specific heat, melting point, heat of fusion, and thermal diffusivity (see Table 1). Therefore, compared to the other two materials, energy deposited in a GaSb target (1) more readily raises the temperature, (2) more rapidly causes it to reach the melting point, (3) more easily induces a high-mobility liquid phase, and (4) remains concentrated longer in the impact region. These observations suggest a final hypothesis: that in the context of irradiation-driven mechanisms, compound targets are more readily disrupted when the local effective “temperature” of the collision cascade reaches temperatures beyond the thermodynamic melting point. We now seek to quantify this idea as recently demonstrated by Bottger *et al.*^69^, and calculate the volume of an “effective melt pool” by means of the following process:

1. We assume energy is deposited with intensity proportional to a Gaussian ellipsoid with vertical and lateral straggle extracted from simulations of each ion/target combination.
2. We identify an ellipsoidal “critical volume,” inside of which the energy is sufficient to raise the target temperature from room temperature to the melting point of the target material.
3. We integrate, over the critical volume, the amount of energy *in excess* of the amount needed to reach the target’s melting temperature. This is the excess energy.
4. We divide the excess energy by the volumetric heat of fusion of the target material.

Mathematically, this process is condensed into the equation


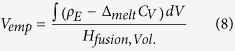


The expression in parenthesis is the deposited energy density 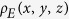 minus the energy density 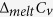 needed to raise the material inside the volume enclosing the collision cascade to its melting point (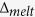 is the difference between room and melting temperatures, and 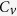 is the volumetric heat capacity). The boundary of the “critical volume” is obtained from the level curve 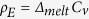, and the integral is performed numerically. Then, division by the volumetric heat of fusion 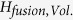 gives the volume of material that would be subject to a phase transition if the material were at equilibrium.

Estimated values of 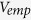 are given in Fig. 6b, which confirms that, despite experiencing lower deposited energy density, GaSb has overwhelmingly larger “effective melt pools” than the other two targets. In fact, the size of 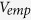 correlates rather well with all of the experimental trends identified in Figs 1 and 2. First, the effective melt pools increase in volume as the ion mass increases, consistent with the emergence of structures as the ion mass increases in Figs 1 and 2. Second, the larger regions of elevated mobility for GaSb are consistent with the much more rapid pattern formation for GaSb observed in Figs 1 and 2, whereas the much smaller high-mobility regions for GaAs and GaP are consistent with slower (or non-existent) pattern formation in these materials.

It is important to acknowledge that for the energies considered here (two orders of magnitude smaller than those considered in ref. 69), actual melting and re-solidification may not occur in the thermodynamic sense. However, the “effective melt pool” described by Eq. (8) quantifies a relationship between material properties and deposited energy density that could potentially serve as the basis for a micro-scale mechanism driving an ion-enhanced mobility. Such mechanisms are sometimes known as athermal processes ([e.g. after Lam & Sigmund^60^) to indicate an indirect correlation to thermodynamic properties, and we believe it is reasonable to assume that cascade effects might similarly affect the dynamics of energetically-driven phase separation, and produce a strongly elevated mobility even in a non-equilibrium setting.

Finally, we acknowledge that the numerical estimates in Fig. 6b do not align perfectly with the experimental observations in Fig. 1. In particular, the effective melt pools for Ar → GaAs and Ar → GaP (where no patterns are observed) are larger than those for Ne → GaSb (where patterns are observed). It is possible that considering the effect of different base thermal diffusivities (i.e., before enhancement by the ion beam), or the re-admission of the assumed small stabilizing effect of ballistic mixing would improve agreement. More broadly, we readily acknowledge that the use of equilibrium thermodynamic reasoning in a highly non-equilibrium setting, though commonly employed as discussed above, is likely to lead only to qualitative insight rather than quantitative predictive power. Nevertheless, and especially in light of these concessions, the correlation between numerical and experimental trends is striking.

### Experimental Observations revisited

Here we briefly revisit two features of the experimental observations above, not previously discussed, which become noteworthy in light of the findings of section IV.

First, we observe that the characteristic scale of the GISAXS satellite peaks of GaSb (for which the greatest variety of patterns exist) are similar for all ions. The q values starts at ~0.19 nm^−1^ (Fig. 2) and decrease after a fluence of 1 × 10^16^ cm^−2^ to ~0.13 nm^−1^ at higher fluences. Because different ions have collision cascades with considerably different size and shape, these observations would be surprising if one assumed that collisional effects induced the structures directly. On the other hand, the independence of these observed properties on the ion mass are plausible if the collision cascade merely provides the mobility for atoms to migrate, either vertically to the surface (as observed by XPS/LEISS), or laterally to enriched phases leading to structures (as observed with GISAXS).

Second, despite the marked difference in pattern formation on GaAs and GaP between Ar and Xe irradiation, the XPS and LEISS results for these targets are not significantly different between irradiation by Ar and irradiation by Xe. Moreover, Ar and Xe irradiation on the different semiconductor surfaces lead to similar element enrichment on the surface. This suggests that the instability is not due to a fundamental difference in the composition or the associated energetic driving force. Again, this observation is reasonable if the most important difference between stable and unstable systems is due to differences in atomic mobility.

The ion-independent value of the wavelength, in particular, is accommodated mathematically within the phase-separation based mechanism. In ref. 23 it was found that a simplified version of the model in the limit 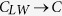 (which is essentially what we observe here in Fig. 4a,b) predicted wavelengths of the form


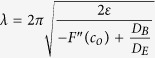


where 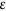 is an energetic penalty for phase boundary formation, and all other symbols have been defined previously. If we continue in our assumption that 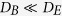 as described above, then this wavelength is at most weakly dependent on the ion mass, consistent with experimental observations.

## Conclusion

By concurrently examining trends in both experimental and numerical studies of ion-induced pattern formation, we have avoided longstanding difficulties impeding direct experimental estimation of model parameters. Instead, we examined the consistency of experimental and numerical trends in light of the requirements imposed by the two most commonly-invoked models of ion beam nanopatterning in III-V semiconductors. Our investigation has shown that the trends are broadly consistent under the assumption that structure formation is caused by a chemical instability such as self-sustained etch masking. Moreover, it suggests that the presence or absence of an instability depends on the strength of an ion-enhanced atomic mobility, and is strongly correlated with the volume of “effective melt pools” created by the impinging ions, which may serve as a micro-mechanism for the mobility enhancement. These findings provide strong support for the consideration of chemically-driven models by the community, and motivate further investigation into the fundamental microscopic physics governing diffusion under ion irradiation.

## Additional Information

**How to cite this article**: El-Atwani, O. *et al.* Ion beam nanopatterning of III-V semiconductors: consistency of experimental and simulation trends within a chemistry-driven theory. *Sci. Rep.* **5**, 18207; doi: 10.1038/srep18207 (2015).

## Figures and Tables

**Table 1:** Relevant thermal properties of the targets used in this study.

	GaSb	GaAs	GaP
Specific Heat [J/(K cc)]	1.40	1.76	1.78
Melting Point [C]	712	1238	1477
Heat of Fusion [kJ/mol] (ref. 70)	44	84	72
Thermal Diffusivity [cm^2^/sec]	0.23	0.31	0.62
